# Proteomic Strategy for the Analysis of the Polychlorobiphenyl-Degrading Cyanobacterium *Anabaena* PD-1 Exposed to Aroclor 1254

**DOI:** 10.1371/journal.pone.0091162

**Published:** 2014-03-11

**Authors:** Hangjun Zhang, Xiaojun Jiang, Wenfeng Xiao, Liping Lu

**Affiliations:** College of Life and Environmental Sciences, Hangzhou Normal University, Hangzhou, Zhejiang, China; University of North Carolina at Chapel Hill, United States of America

## Abstract

The cyanobacterium *Anabaena* PD-1, which was originally isolated from polychlorobiphenyl (PCB)-contaminated paddy soils, has capabilities for dechlorinatin and for degrading the commercial PCB mixture Aroclor 1254. In this study, 25 upregulated proteins were identified using 2D electrophoresis (2-DE) coupled with matrix-assisted laser desorption/ionization time of flight mass spectrometry (MALDI-TOF MS). These proteins were involved in (i) PCB degradation (i.e., 3-chlorobenzoate-3,4-dioxygenase); (ii) transport processes [e.g., ATP-binding cassette (ABC) transporter substrate-binding protein, amino acid ABC transporter substrate-binding protein, peptide ABC transporter substrate-binding protein, putrescine-binding protein, periplasmic solute-binding protein, branched-chain amino acid uptake periplasmic solute-binding protein, periplasmic phosphate-binding protein, phosphonate ABC transporter substrate-binding protein, and xylose ABC transporter substrate-binding protein]; (iii) energetic metabolism (e.g., methanol/ethanol family pyrroloquinoline quinone (PQQ)-dependent dehydrogenase, malate-CoA ligase subunit beta, enolase, ATP synthase β subunit, F_O_F_1_ ATP synthase subunit beta, ATP synthase α subunit, and IMP cyclohydrolase); (iv) electron transport (cytochrome b_6_f complex Fe-S protein); (v) general stress response (e.g., molecular chaperone DnaK, elongation factor G, and translation elongation factor thermostable); (vi) carbon metabolism (methanol dehydrogenase and malate-CoA ligase subunit beta); and (vii) nitrogen reductase (nitrous oxide reductase). The results of real-time polymerase chain reaction showed that the genes encoding for dioxygenase, ABC transporters, transmembrane proteins, electron transporter, and energetic metabolism proteins were significantly upregulated during PCB degradation. These genes upregulated by 1.26- to 8.98-fold. These findings reveal the resistance and adaptation of cyanobacterium to the presence of PCBs, shedding light on the complexity of PCB catabolism by *Anabaena* PD-1.

## Introduction

Persistent organic pollutants (POPs), such as polychlorinated biphenyls (PCBs) and organochlorine pesticides, are ubiquitous chloroorganic chemicals in the environment. PCBs were first manufactured in the United States in 1929 [1]. They are a complex class of hydrophobic, lipophilic chemicals that often slowly decompose and metabolize in natural systems [2]. Although PCB production has been banned in 1979, they still pose environmental and human risks in areas of hotspot contamination because of their stable physicochemical properties, hydrophobic properties, and high toxicity [3], [4]. Therefore, cleaning residual PCB-contaminated environments has elicited significant research attention in the last few decades.

Several physical, chemical, and biological methods are available for PCB degradation [5]. Biological methods for PCB degradation have been extensively studied and considered crucial for the biodegradation of PCBs because of their low environmental impact and economic advantage compared with physicochemical methods [6]. PCB degradation is exhibited by several bacteria and fungi, such as *Pseudomonas pseudoalcaligenes* KF707 [7], *Burkholderia cepacia* LB400 [7], *Sinorhizobium meliloti* [8], *Hydrogenophaga* sp. strain IA3-A [9], *Pleurotus ostreatus* [10], and *Ceriporia* sp. ZLY-2010 [11].

Microalgae and cyanobacteria are common species in the natural environment. They can act as distinctive biological agents for organic pollutant degradation [12] and can be used to degrade organic pollutants. The use of microalgae and cyanobacteria has become a new method for POP degradation in recent years. Kotzabasis et al. [13], [14] have reported that *Scenedesmus obliquus* can biodegrade dichlorophenols. *Chlorella fusca* var. *vacuolata* can remove 23% of 2,4-dichlorophenol after 4 d [15]. The microalga *Cyclotella caspia* can degrade the aromatic pollutant nonylphenol [16]. The *Anabaena flos-aquae* strain 4054 can decompose endocrine-disrupting pollutants, such as phthalate esters [17]. *Anabaena azotica*, another common cyanobacterium, can effectively degrade the organochlorine pesticide γ-hexachlorocyclohexane (lindane) [18]. Therefore, cyanobacterial species with degradation functions may be a potential choice for PCB degradation.

The survival of wild-type microorganisms with degradation function may be limited by adverse environmental conditions, leading to reduced degradation efficiency [19]. Fortunately, genetically engineered microbes can enhance degradation efficiency by enhancing the activity of key enzymes via genetic engineering [20]. The genes and proteins in microbes have important functions in organic pollutant degradation [21]–[23]. The molecular mechanism by which microorganisms degrade PCB has also been explored by utilizing proteomic technologies, including 2D electrophoresis (2-DE) and matrix-assisted laser desorption/ionization time-of-flight mass spectrometry (MALDI-TOF MS) [24]. The enzymes in the PCB degradation pathway include biphenyl 2,3-dioxygenases, *cis*-2,3-dihydro-2,3-dihydroxybiphenyl dehydrogenase, 2,3-dihydroxybiphenyl 1,2-dioxygenases, 2-hydroxy-6-phenyl-6-oxohexa-2,4-dienoate hydrolases, 2-hydroxypenta-2,4-dienoate hydratase, 4-hydroxy-2-oxovalerate aldolase, and acetaldehyde dehydrogenase [25]. Exposure to aromatic compounds stimulates metabolic enzymes and other polypeptides in microorganisms. Proteins involved in energy metabolism and substrate transport are upregulated during the degradation of aromatic pollutants and organochlorine pesticides by various microorganisms [26]–[28].

Our previous study produced encouraging results in PCB biodegradation by cyanobacteria. The cyanobacterium *Anabaena* PD-1 was originally isolated from PCB-contaminated paddy soil and exhibited strong ability to degrade PCB congeners (data not shown). However, proteomic analyses of cyanobacterial responses to stressors have mainly focused on salt [29], acid [30], and arsenic [31]. Limited information is available on the cyanobacterial catabolism of PCBs and the responses of cyanobacteria to PCBs [12]. Therefore, the key enzymes involved in PCB degradation need to be identified and the degradation mechanism should be explored to gain important information on the construction of genetically engineered cyanobacteria. Such genetically engineered cyanobacteria may achieve the same or higher PCB degradation efficiency compared with laboratory conditions.

In this study, we separated differentially expressed proteins through 2-DE and identified polypeptides through MALDI-TOF tandem mass spectrometry (MS/MS). Protein information was obtained from the NCBInr database through its Mascot search engine. Real-time PCR was utilized to analyze the genes encoding for highly expressed key proteins during PCB degradation in *Anabaena* PD-1 cells. The present contribution can provide new insights into the biodegradation of PCBs by *Anabaena* PD-1 and into the construction of genetically engineered PCB-degrading cyanobacterial species.

## Materials and Methods

### Strain *Anabaena* PD-1 and culture conditions

*Anabaena* PD-1, a PCB-tolerant strain, was isolated from PCB-contaminated paddy soil in Taizhou, Zhejiang, China (No specific permissions were required for the sampling locations and activities. The field studies did not involve endangered and protected species. The sampling site in the study is located at Latitude 28°32′N Longitude 121°27′E.). *Anabaena* PD-1 cells were grown at 25°C, 998 lux, in BG-11 medium [32], under discontinuous illumination (light ∶ dark  = 12 h ∶ 12 h). Cyanobacterial cells in their exponential phase were cultivated with and without Aroclor 1254 (2 mg/L) for 30 d for the PCB-degrading experiment.

### Preparation of cellular proteins

The cells were cultured for 30 d and ground to powder with liquid nitrogen. Subsequently, 10 mL of cooled acetone containing 10% trichloroacetic acid and 0.07% DTT was added to 1 g of sample powder at −20°C for 1 h. The deposit was collected after centrifugation at 15000 g for 15 min at 4°C. Cooled acetone containing 0.07% DTT was then added to the deposit at −20°C for 1 h. After another centrifugation at 15000 g for 15 min at 4°C, the deposit was collected and dried with a vacuum freeze dryer. The powder was dissolved in a lysis solution [9 M urea, 4% CHAPS, 1% DTT, 1% IPG buffer (GE Healthcare)] at 50 μL: 1 mg, dissolved at 30°C for 1 h, and then centrifuged again at 15000 g for 15 min at room temperature. The concentrations of the protein extracts were determined with the Bradford method [33]. The extracts were then stored at −80°C for isoelectric focusing electrophoresis (I FE).

### 2-DE

#### IFE

Samples containing 200 μg of proteins were mixed with a fresh rehydration buffer [9 M urea, 4% CHAPS, 1% DTT, 1% IPG buffer (GE Healthcare), trace amount of bromophenol blue] to form a 450 μL mixture. DryStrip (GE Healthcare, 24 cm, pH 3 to 10, NL) was obtained from a −20°C freezer, placed at room temperature for 10 min, added to the protein sample in the strip holder, and then subjected to IEF according to the following protocol: rehydration at 50 V (12 h), 500 V (1 h), 1000 V (1 h), 10000 V (1 h), and 10000 V (10 h). All steps were controlled at 50 μA/gel at 20°C.

### Equilibration and SDS-PAGE

After IEF, the strip removed from the strip holder was incubated in equilibration buffer 1 [6 M urea, 30% glycerol, 2% SDS, 50 mM Tris-HCl (pH 8.8), 1% DTT, and trace amount of bromophenol blue] for 15 min and then in equilibration buffer 2 [6 M urea, 30% glycerol, 2% SDS, 50 mM Tris-HCl (pH 8.8), 2.5% iodoacetamide, and trace amount of bromophenol blue] for 15 min. After the strip was rinsed with SDS-PAGE buffer for 10 s, a sealing solution was added to the surface of the SDS-PAGE gel. The gel was then moved to the electrophoresis apparatus for electrophoresis at the following parameters: 100 V, 15°C, 45 min, followed by 200 V for 6 h to 8 h (Ettan DALTsix system). The gels were then stained with silver nitrate according to the method described by Shevchenk et al. [34].

### Gel visualization, scanning, and analysis

The gels were visualized by silver staining for analysis. The stained gels were scanned by an image scanner (GE Healthcare, USA) at a resolution of 300 dots per inch. All gel images were processed by spot detection, volumetric quantification, and matching with PDQuest 8.0 software. The differences in protein content between the treatment and control groups were calculated as fold ratios. A fold change ≥2.0 or ≤0.5 was utilized to differentially select protein spots.

### In-gel digestion and MS analysis

The proteins were digested by 50% ceric ammonium nitrate for 5 min, followed by 100% ACN for 5 min, and then rehydrated in 2 μL to 4 μL trypsin (Promega, Madison, USA) solution (20 μg/mL in 25 mM NH_4_HCO_3_) for 30 min. A 20 μL cover solution (25 mM NH_4_HCO_3_) was then added for 16 h of digestion at 37°C. Afterward, the supernatants were transferred into another tube, and the gels were extracted once with a 50 μL extraction buffer (67% ACN and 5% TFA). The peptide extracts and supernatant of the gel spot were combined and completely dried. The samples were analyzed with an ABI 4800 MALDI-TOF/TOF Plus mass spectrometer (Applied Biosystems, Foster City, USA). Data were obtained with a positive MS reflector. CalMix5 standard was utilized to calibrate the instrument (ABI4800 calibration mixture). MS and MS/MS data were integrated and processed with GPS Explorer V3.6 (Applied Biosystems, USA) with default parameters. According to combined MS and MS/MS spectra, the proteins were successfully identified at 95% or higher confidence interval of their scores in the MASCOT V2.1 search engine (Matrix Science, London, UK). The following search parameters were used: NCBInr database; trypsin as the digestion enzyme, one missed cleavage site, fixed modifications of carbamidomethyl (C), partial modifications of acetyl (protein N-term), and oxidization (M); 200 ppm for precursor ion tolerance and 0.5 Da for fragment ion tolerance.

### RNA preparation and real time-PCR

Total RNA was extracted from 50 mg to 80 mg of cyanobacterial cell pellets (OD_680_ = 0.38) using TRIzol Reagent (Invitrogen, Carlsbad, CA, USA). RNA was then purified by removal of genomic DNA contaminants using an RNase-free DNase I kit (Invitrogen) and verified by determination of 260/280 nm ratios and 1% agarose–formaldehyde gel electrophoresis with ethidium bromide staining. Total RNA was subjected to cDNA synthesis using a NuGEN OvationW Prokaryotic RNA-Seq System according to the manufacturer's instructions (Haoji Biotechonlogy Hangzhou, China). RT-PCR was carried out with a multiplex real-time PCR detector (BioRad, USA). The reaction mixture included a Power Master Mix (Invitrogen), 0.5 mM of the primers, MilliQ water, and 1 mL of cDNA. The thermal cycling program was as follows: predegeneration at 95°C for 1 min, followed by 40 cycles of denaturation at 95°C for 10 s, and then annealing at 62°C for 25 s. Primer sequences for 3-chlorobenzoate-3,4-dioxygenase, cytochrome b_6_f complex Fe-S protein, transporter proteins, and energetic metabolism proteins are shown in Table 1.

**Table 1:** Primers used for the quantitative real-time polymerase chain reaction in this study.

Gene name	Primer Sequence (5′-3′)	GenBank Accession #^a^	Size (bp)
3-chlorobenzoate-3,4-dioxygenase gene	F: GCCCCAAATCAGAAACTACCAR: CCATCACCGGGAAATAACCAA	­	89
Cytochrome b6f complex Fe-S subunit gene	F: TTAAATGCCCTTGCCACGGTTCTCR: AGCGTGACTCAAAGCCAGAGACTT	NC_007413	89
ABC transporter substrate-binding protein gene	F: GCTGCATCGCAACCAATCAAAR: GGTATATCTGCCAGCCGGAACA	NC_007413	120
Enolase gene	F: TTGCCTGTGCCTTTAATGAACGTR: AAGCCTTTGTCATGCAGCACTTC	­	170
Porin gene	F: CCACAACAAAGCTGCAAGGACAR: TGAACAAGGTATCTCGCCCAGTAAA	NC_007413	159
fraH gene	F: ATGTTGATGTTTCCGGCTTTGCR: GGTCTGAGGCGGTGTCTATTGC	NC_007413	163
methanol dehydrogenase gene	F: TTAGCAGAGGTGGCAGAATTACGAR: CCCGTGGACTGACACCGAGA	NC_007413	133
malate-CoA ligase subunit beta gene	F: TTTGCGTAATTGGCATACCAGATAAR: TGGGGGTTACGGGGTAAGGTATT	NC_007413	175

a Primers with accession numbers belong to a gene cluster. Primers without accession numbers were designed according to the reference [35].

### Statistical analysis

The statistical differences of the experimental data were determined using one-way ANOVA followed by two-sided Dunnett's t-test. Statistical tests were conducted using SPSS11.0, and the statistical significance values were defined as *P<0.05 and **P<0.01. All data were expressed as mean±standard deviation (S.D.).

## Results

### Protein expression patterns of *Anabaena* PD-1 exposed to Aroclor 1254

**Figure 1:**
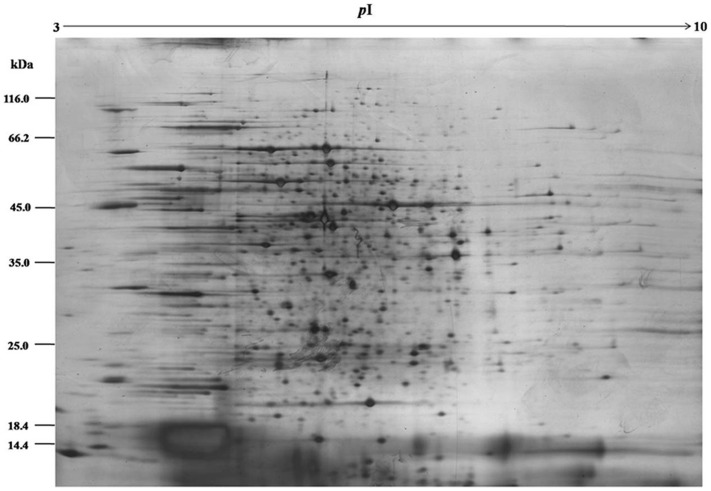
2D gel maps for differentially expressed proteins in *Anabaena* PD-1 cells in the control group.

**Figure 2:**
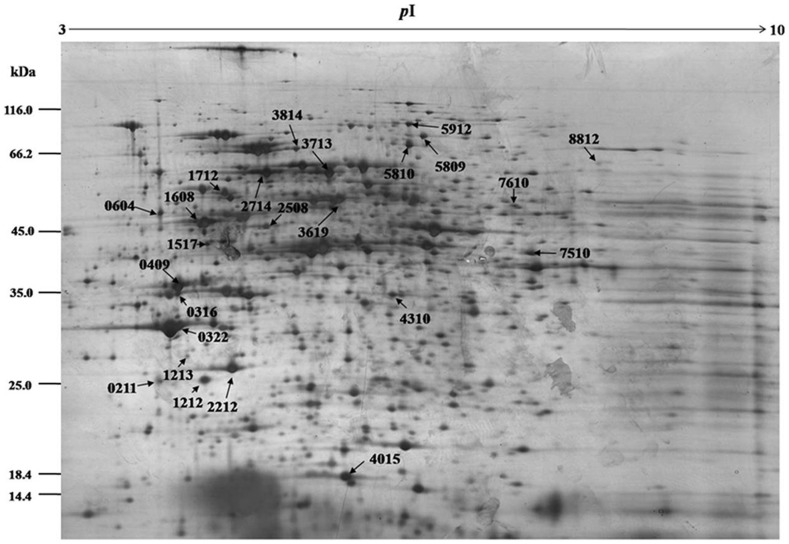
2D gel maps for differentially expressed proteins in *Anabaena* PD-1 cells in the Aroclor 1254 degradation group. Arrow-directed spots are upregulated proteins in PCB degradation by *Anabaena* PD-1. The detailed information of upregulated proteins are listed in Table 2.

**Figure 3:**
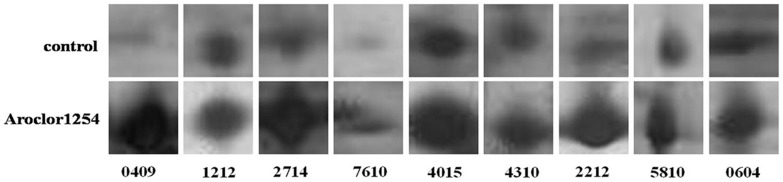
Upregulated proteins of *Anabaena* PD-1 exposed to Aroclor 1254 for 30 d. 0409-ABC transporter substrate-binding protein, 1212-amino acid ABC transporter substrate-binding protein, 2714-methanol/ethanol family PQQ-dependent dehydrogenase, 7610-bifunctional phosphoribosylaminoimidazolecarboxamide formyltransferase/IMP cyclohydrolase, 4015-cytochrome b6-f complex iron-sulfur subunit, 4310-Rieske FeS protein, 2212-ureidoglycolate lyase, 5810-nitrous oxide reductase, and 0604-putative heterocyst to vegetative cell connection protein.

Total proteins extracted from the PCB-treated and reference cyanobacterial samples were separated through 2-DE. Most proteins were located between pH 4.0 and 6.8 and then weighed 17 kDa to 96 kDa (Figure 1 and Figure 2). Exposure to Aroclor 1254 diversified the overall protein expression patterns of *Anabaena* PD-1. A total of 25 protein spots were up-regulated (multiple changes were twice greater; Student's t-test, *P*<0.05). Twenty-six differentially expressed proteins were identified through MALDI-TOF MS/MS. Detailed information on these proteins is summarized in Table 2. The proteins involved in PCB-degradation, transport processes, energy metabolism, and electron transport are shown in Figure 3.

**Table 2:** Upregulated proteins in *Anabaena* PD-1 cells during PCB degradation.

Progein function	Spot No.	Identified protein	Accession No.	Amino acid sequences	Sequence Coverage	Score	*p*I		Molecular Mass (kDa)		Fold change
							Expected	Observed	Expected	Observed	
**transportation**	0409	ABC transporter substrate-bind ing protein	gi|222149178	GNTGPQAPDVIDVGLSFGPAAK	6%	183	5.67	4.51	38.9	36.5	734.5
	1212	amino acid ABC transporter substrate-binding protein	gi|222149574	TPYGQGLADETKK	11%	283	6.04	4.68	39.0	27.3	623.4
	1712	peptide ABC transporter substrate-binding protein	gi|328542580	QAIDNLVFAITPDAAVR	3%	123	5.11	4.80	59.0	58.8	381.6
	0316	putrescine ABC transporter periplasmic putrescine-binding protein	gi|117617823	QIKAGVFQK	2%	61	6.04	4.50	40.3	35.7	218.2
	1213	phosphonate ABC transporter substrate-binding protein	gi|493775881	GFTEVNVDFYKPIIEAR	13%	187	4.92	4.65	32.2	29.0	15.7
	1517	urea short-chain amide or branched-chain amino acid uptake ABC transporter periplasmic solute-binding protein	gi|39936731	ELNSILFYPVQYEGEESER	10%	239	7.63	4.72	48.2	45.3	12.6
	0322	phosphonate ABC transporter substrate-binding protein	gi|497516437	FCEGVGKNTIDIANASR	4%	135	4.24	4.54	37.4	32.6	9.8
	0211	xylose ABC transporter substrate-binding protein	gi|517198395	AQNEGIPVVGYDR	3%	91	5.48	4.28	36.3	28.5	7.3
**Energetic metabolism**	2508	enolase	gi|493227560	VNQIGSLTETLDAVETAHK	16%	445	5.11	4.99	45.3	48.6	239.9
	7610	bifunctional phosphoribosylaminoimidazolecarboxamide formyltransferase/IMP cyclohydrolase	gi|17230585	TAAAAGISAIVQPGGSLR	17%	401	5.56	6.24	54.3	54.8	23.0
	8812	ATP synthase beta subunit	gi|9909749	AHGGYSVFAGVGER	16%	67	6.51	6.72	15.6	68.1	10.4
	3619	FoF1 ATP synthase subunit beta	gi|154251918	AHGGYSVFAGVGER	17%	539	4.95	5.37	51.3	55.4	7.6
	3713	ATP synthase subunit alpha	gi|17134983	EAYPGDVFYIHSR	13%	290	5.11	5.31	54.4	63.4	5.01
**Electron transport**	4015	cytochrome b_6_f complex iron-sulfur subunit	gi|17229945	CPCHGSQYDATGK	21%	246	5.31	5.42	19.2	20.0	11.5
	4310	Rieske-FeS protein	gi|14272374	FLESHNVGDR	5%	63	5.31	5.64	19.2	35.5	9.56
**Carbon metabolism**	2714	methanol/ethanol family PQQ-dependent dehydrogenase	gi|170743819	QDPNVIPVMCCDTVNR	4%	129	6.23	5.00	65.5	64.0	499.2
	7510	malate-CoA ligase subunit beta	gi|227823162	GGLAYSPEQAAYR	3%	107	5.16	6.31	43.0	43.1	449.1
**Nitrogen metabolism**	2212	ureidoglycolate lyase	gi|518240147	YIDESNALDHVAGYCVINDVSER	8%	111	4.89	4.88	30.6	29.4	172.8
	5810	nitrous oxide reductase	gi|226346680	ILTEGLLPETR	6%	83	5.64	5.72	37.4	74.0	9.7
**Transmembrane protein**	1608	porin; major outer membrane protein	gi|17130179	VNNADIVDTNTTLGVR	15%	255	4.61	4.69	54.4	50.1	379.9
	0604	putative heterocyst to vegetative cell connection protein	gi|556608	IPPDVDVSGFANSEIVSR	31%	473	4.46	4.38	29.9	52.5	192.9
**Other proteins**	5912	elongation factor G	gi|516958835	LNIIDTPGHVDFTIEVER	7%	227	5.08	5.72	76.1	83.4	8.1
	5809	100RNP protein	gi|1588265	AHGSALFTR	1%	68	5.89	5.79	85.2	78.1	7.4
	1216	hypothetical protein	gi|518291170	NLGLVDPNSTSGNNVPR	8%	217	9.27	4.82	33.9	28.3	4.9
	3814	molecular chaperone DnaK	gi|519030894	DAGLSAGQIDEVVLVGGMTR	7%	227	4.92	5.15	67.9	72.4	4.7

### Real time-PCR analysis of upregulated protein-encoding genes in *Anabaena* PD-1 cells

**Figure 4:**
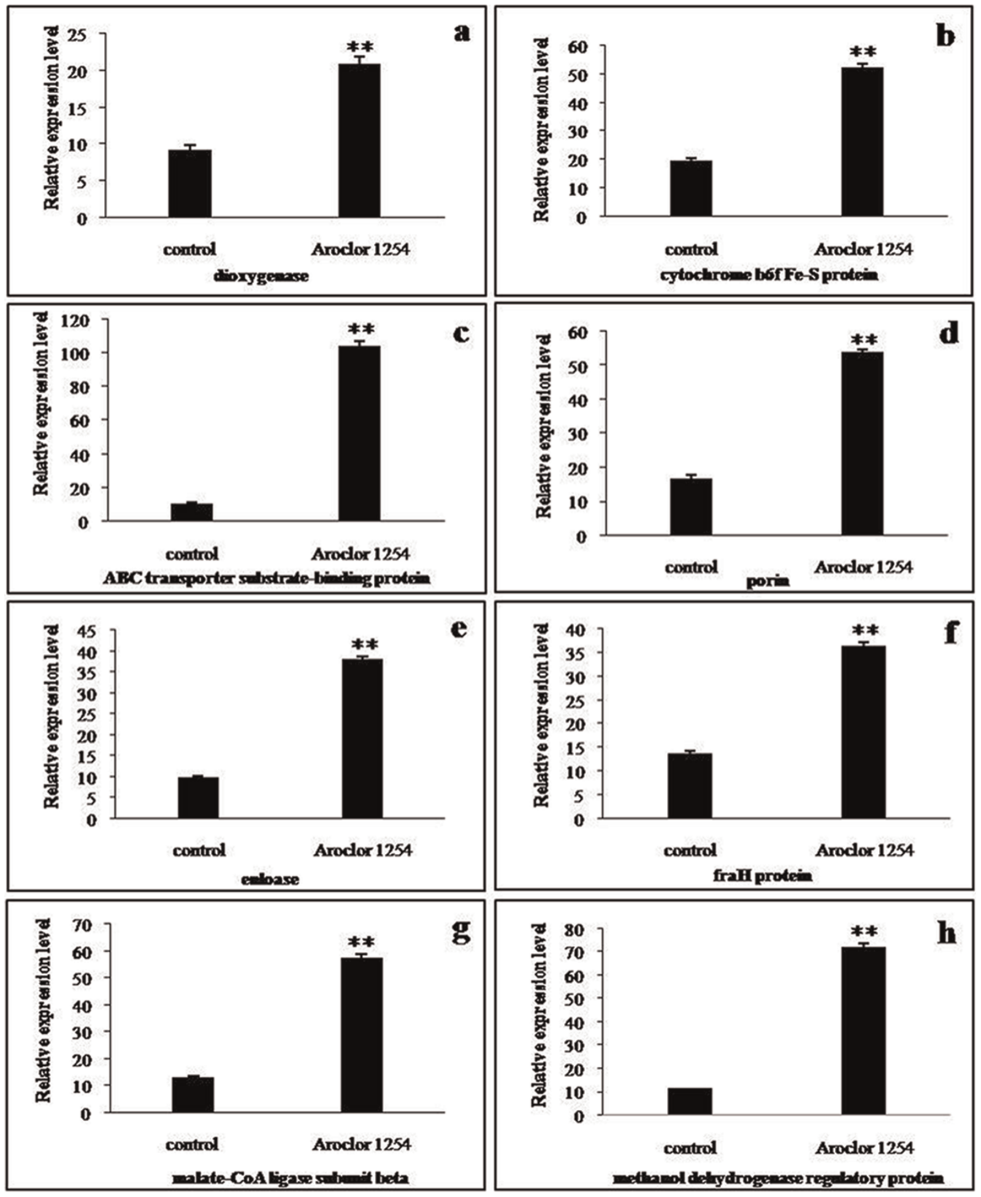
Gene expression levels in control and PCB-treated groups. Expression levels of genes encoding for (a) dioxygenase, (b) cytochrome b6f Fe-S protein, (c) ABC transporter substrate-binding protein, (d) porin, (e) enloase, (f) fraH, (g) malate-CoA ligase, and (h) methanol dehydrogenase. Data represented the mean ± SD, and significant difference from the control group was determined by *p<0.05 and **p<0.01.

The expression levels of eight genes in the PCB-degradation groups were significantly upregulated compared with those in the control groups (*P*<0.01). The results are presented in Figure 4. Genes encoding for dioxygenase were upregulated by 1.26-fold. The cytochrome b_6_f complex Fe-S protein-encoding gene upregulated by 2.64 fold. The ABC transporter substrate-binding protein gene expression level in the PCB-treated groups was 9.98-fold of that in the control groups. Transmembrane protein-encoding genes (i.e., heterocyst to vegetative cell connection protein and porin genes) were upregulated by 2.66- and 3.19-fold, respectively. Enolase gene expression level was upregulated by 2.88-fold compared with the control groups. The upregulation levels of the malate-CoA ligase subunit beta gene and methanol dehydrogenase gene were 3.40 and 5.22, respectively.

## Discussion

*Anabaena* PD-1 isolated from PCB-contaminated paddy soil in South Zhejiang, China, efficiently degrades PCBs. Rodrigues et al. [36] suggested that *Burkholderia xenovorans* LB400, one of the most extensively studied PCB-degrading bacteria, can degrade 57% of Aroclor 1242 in 30 d. Singer et al. [35] combined *Arthrobacter* sp. strain B1B with *Ralstonia eutrophua* H850 to degrade PCB mixtures and achieved a maximum degradation rate of 59% in over 18 weeks. These bacterial species are typical PCB degraders. However, the application of these species to the bioremediation of PCB-contaminated paddy soils is limited because of their specific living conditions. By contrast, *Anabaena* PD-1, an associated cyanobacterial species in paddy soils, adapts to the condition in paddy soils very well. This condition is one of the essential requirements for this functional species to degrade PCBs in contaminated paddy soils. Thus, *Anabaena* PD-1 may be an excellent choice for the remediation of PCB-contaminated paddy soils.

**Figure 5:**
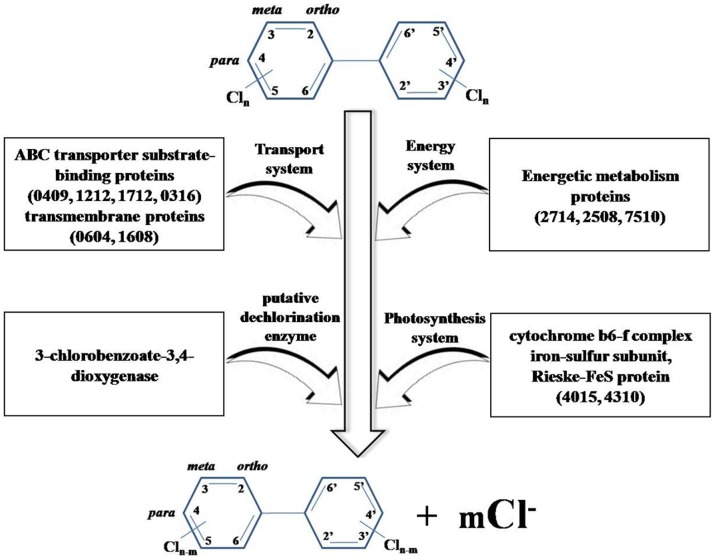
Hypothetic scheme of PCB degradation by Anabaena PD-1. Numbers in brackets present the different proteins in Table 2.

This study is the first to report on the proteome files for *Anabeana* PD-1 in the PCB degradation and control groups and the RT-PCR gene data of upregulated proteins during PCB degradation. We synthesized the information from the protein and gene levels of the PCB-degrading cyanobacterial species *Anabaena* PD-1 and proposed a putative scheme to demonstrate the possible biodegradation mechanism of PCBs by *Anabaena* PD-1 (Figure 5). We assumed that 3-chlorobenzate-3,4-dioxygenase, transporter proteins, electron transport proteins, transmembrane proteins, and energetic metabolism proteins and genes encoding for the said proteins have important functions during PCB dechlorination by *Anabaena* PD-1. As a complex bioreaction, PCB degradation is involved with the transport system, energy system, and photosynthetic system in *Anabaena* PD-1 cells.

Organic pollutant stressors can generally change the cell membrane structure and function [36] and exert general stress on cells. The different (chlorinated) aromatic compounds induced the overexpression of different proteins (Table 3). Stressors with chlorinated structures can enhance the expression of stress protein DnaK, ABC transporter, and enolase, whereas pollutants without chlorinated structures can increase the expression of branched-chain amino acid uptake ABC transporter periplasmic solute-binding proteins and elongation factor G. This difference in protein expression may be attributed to the various structures of the organic stressors. The upregulated proteins with different functions can provide new insights into the adaptation of *Anabaena* PD-1 to the presence of PCBs.

**Table 3:** Comparison of induced proteins by (chlorinated) aromatic compounds in *Anabaena* PD-1 and other microorganisms.

Induced proteins in this study	Organic pollutant stressors	Microorganisms	Approaches	References
molecular chaperone DnaK	quinclorac	*Burkholderia cepacia* WZ1	2-DE, MALDI-TOF MS/MS	[37]
	3,4-dichloroaniline	*Variovorax* sp. WDL1	2-DE, MALDI-MS(/MS)	[38]
	4-chlorophenol	*Pseudomonas putida*	2-DE, MALDI-TOF MS	[39]
	4-chloronitrobenzene	*Comamonas* sp. strain CNB-1	2-DE, MALDI-TOF MS	[40]
	benzoate	*Pseudomonas putida* P8	2-DE, MALDI-TOF MS	[28]
ABC transporter substrate binding protein	2,4-dichlorophenoxy acetic acid	*Corynebacterium glutamicum*	2-DE, MALDI-TOF MS/MS	[41]
	phenol	*Pseudomonas putida* KT2440	2-DE, MALDI-TOF MS	[42]
	benzoate	*Pseudomonas putida* P8	2-DE, MALDI-TOF MS	[28]
	benzoate & succinate	*Pseudomonas putida* KT2440	iTRAQ, 1-DEMudPIT	[43]
translation elongation factor TS	4-chloronitrobenzene	*Comamonas* sp. strain CNB-1	2-DE, MALDI-TOF MS	[40]
enolase	4-chloronitrobenzene	*Comamonas* sp. strain CNB-1	2-DE, MALDI-TOF MS	[40]
branched-chain amino acid uptake ABC transporter periplasmic solute-binding protein	benzoate	*Pseudomonas putida* P8	2-DE, MALDI-TOF MS	[28]

In this study, 3-chlorobenzoate-3,4-dioxygenase, a new enzyme that may be involved in PCB degradation by *Anabaena* PD-1, belongs to the family of Rieske protein family (http://pfam.janelia.org/family/PF00355.). Thus, this dioxygenase is closely related to cytochrome b_6_f complex Fe-S protein, another typical Rieske protein [44]. The gene encoding for 3-chlorobenzoate-3,4-dioxygenase is named *cbaA* [45]. This enzyme has an important function in the degradation of pollutants belonging to the toluene/biphenyl family [46]. Thus, we believe that 3-chlorobenzoate-3,4-dioxygenase may be closely related to the direct biodegradation of Aroclor 1254 by *Anabaena* PD-1. Nevertheless, the mechanism by which 3-chlorobenzoate-3,4-dioxygenase participates in PCB dechlorination in *Anabaena* PD-1 cells remains intriguing.

The PCB-treated and reference gels indicated that protein spots (4015, 4310) are significantly upregulated. These spots are *petC* gene products or PetC proteins that were first discovered and isolated by Rieske et al. [47]. Thus, the proteins are also called Rieske proteins. PetC protein is a subunit of cytochrome bc1 and cytochrome b_6_f complexes. Two to four genes of the *petC* gene family are found in the cyanobacterial cells of *Nostoc* sp. PCC 7120 [48]. PetC protein is an essential protein for the functioning of the cytochrome b_6_f complex. Cytochrome b_6_f complex has an important function in the aerobic photosynthetic electron transport chain reaction center [49]. The reaction center is involved in energy metabolism and electron transfer. Organic pollutants, such as PCBs, usually act as electron acceptors. Thus, as a key enzyme-mediating electron transporter, PetC protein upregulation may influence the biodegradation of PCBs in cyanobacterial cells. The changes in the expression levels of cytochrome b_6_f complex Fe-S protein confirmed this inference (Figure 4b). Comparing Figure 1 and Figure 2, the expression of proteins 0409, 1212, 1712, 0316, 1213, 1517, 2508, 0322, and 0211 were significantly enhanced. These proteins belong to the ABC transporter family and are ABC transporter substrate-binding proteins (0409, 1212, and 1712), ABC transporter periplasmic putrescine-binding protein (0316), phosphonate ABC transporter substrate-binding protein (1213, 0322), urea short-chain amide or branched-chain amino acid uptake periplasmic solute-binding protein (1517), and xylose ABC transporter substrate-binding protein (0211). The ABC transporter family comprises proteins that can transport ions, saccharides, lipids, and heavy metals across membranes [42]–[43], [50]. The upregulation of these transport proteins indicates that they are likely to participate in PCB uptake or metabolite efflux. The upregulation of ABC transport proteins in *Pseudomonas putida* P8 suggests that the increased transport of amino acids is a cellular response to external stress [27]. The degradation of PCBs by the nitrogen-fixing species *Anabaena* PD-1 consumes energy. Thus, cyanobacterial cells bind to synthesize large amounts of ATP, thereby increasing the substrates required for ATP synthesis. The upregulated expression of transport proteins in algal cells may be due to the increased need for transporting ATP synthesis substrates. Several other studies have detected the efflux of organic solvents induced by transporters in *Pseudomonas putida* in response to aromatic and aliphatic solvents and alcohols [51].

The upregulation of enzymes involved in energetic metabolism have also been observed and found to be consistent with the extra-energetic requirements of cells that trigger several energetically expensive short-term adaptation mechanisms to survive and adapt to the toxicity of PCBs. Enolase (2508) and ATP synthase β subunit (8812) are enzymes involved in energy synthesis and metabolism. ATP synthase β subunit is involved in ATP synthesis under the conditions of a transmembrane proton gradient. The upregulation of this enzyme benefits the cells exposed to toxic organic compounds that can cause membrane lesions [52]. Enolase participates in glycolysis, which converts 2-phosphoglycerate to phosphoenolpyruvate [26]. Enolase consumes energy for cells to actively transport organic compounds to the intracellular space or transport such compounds from the intracellular space to the extracellular space [53]. Pérez-Pantoja [54] reported that the enolase superfamily does not participate in aromatic pollutant degradation by *Mycobacterium smegmatis*. Nevertheless, we still believe that enolase may be indirectly involved in PCB degradation by *Anabaena* PD-1 because of the significant upregulation in both protein (Figure 3) and gene levels (Figure 4). The upregulation of ATP synthase β subunit and F_O_F_1_-ATP synthase β subunit (3619) indicates that the ATP consumption of nitrogen-fixing cyanobacterial cells exposed to Aroclor 1254 significantly increases because cyanobacterial cells actively transport PCB molecules to the extracellular space to protect themselves from the toxicity of PCBs. Another possibility is that cyanobacterial cells consume energy to transport PCB molecules to the intracellular space for degradation. Stress proteins are consistently upregulated to protect cells from organic pollutant stressors. Therefore, the upregulation of several proteins involved in polypeptide folding and synthesis is expected [55]. Molecular chaperone DnaK (3814) is a member of the HSP 70 (heat shock protein weighing 70 kDa) family. HSPs are highly conversed proteins that can assist in the refolding or hydrolysis of abnormal proteins [56]. The expression of these proteins in cells is usually upregulated under several stress conditions to protect cells [57]–[58]. Aromatic compounds can disrupt the synthesis of cell membranes, produce unfolded membrane proteins, and activate the stress response of membrane proteins [59]. Thus, the presence of PCBs causes stress to nitrogen-fixing cyanobacteria. The upregulated expression of chaperone proteins indicates that *Anabaena* PD-1 cells produce several stress responses to PCBs probably to protect themselves from the toxicity of PCBs.

In summary, changes in the proteome of *Anabaena* PD-1 cells during PCB degradation, gene encoding, and upregulation of proteins were observed for the first time. Twenty-five upregulated proteins were successfully identified. The *cbaA* gene encoding for 3-chlorobenzoate-3,4-dioxygenase was upregulated. Electron transport protein *petC* gene product was upregulated as well. The largest group of proteins enhanced by PCBs consists of the substrate-binding proteins of ABC transporters and proteins involved in energy metabolism. Although stress protein DnaK and several other proteins, such as elongation factors, compose a small portion of the upregulated proteins, they may still have important functions in the adaptation of *Anabaena* PD-1 to PCB and the degradation of PCBs by *Anabaena* PD-1. Thus, more studies should be conducted to identify the metabolites of PCBs and explore the PCB degradation pathway by *Anabaena* PD-1.
